# Genomic analysis of phylogroup D *Escherichia coli* strains using novel *de-novo* reference-based guided assembly

**DOI:** 10.1038/s41597-023-02444-0

**Published:** 2023-09-01

**Authors:** Manisha Aswal, Neelja Singhal, Manish Kumar

**Affiliations:** Department of Biophysics, University of Delhi South Campus, New Delhi, India

**Keywords:** Genome assembly algorithms, Gene ontology

## Abstract

*Escherichia coli* are highly diverse bacteria with different pathogenic types, serotypes and phylogenetic types/phylotypes. In recent years, infections with *E. coli* have increased worldwide and so has the emergence of antibiotic resistant strains. In the present study we have assembled, annotated and analysed genome sequences of three strains of the phylogroup D of *E. coli*. These strains were isolated from the river Yamuna, a prominent anthropogenic urban river of northern India. These strains showed varied antibiotic susceptibilities, one was susceptible to all the antibiotics tested except ampicillin while of the other two, one was multi-β-lactam resistant and the other was multi-drug resistant (resistant to multiple β-lactams, fluoroquinolones and kanamycin). The short-sequence reads were assembled into contigs using the *de-novo* approach and further, scaffolding of contigs was performed by using the best reference genome for a particular isolate which resulted in a significant increase in the N_50_ value of each assembly. The bioinformatics assembly approach used in this study could be easily applied to study other bacterial genomes.

## Background & Summary

*Escherichia coli* are highly diverse bacteria with different pathogenic types, serotypes and phylogenetic types/phylotypes^1,2^. They were earlier believed to be the inhabitants of the lower intestinal tract of human beings and warm-blooded animals which reached the environment through the faecal discharge and waste-water treatment plants^3^. However, recent studies have shown that besides host, *E. coli* can successfully survive in various ecological niches like soil, water and plantations^3–8^. Genomic and phylogenetic studies have identified divergent lineages of *E. coli* which can survive in various ecological niches, well-adapted to a non-host lifestyle^9^. Commensal strains of *E. coli* (gut-associated, non-pathogenic) mostly belong to phylogroups A and B1 while the pathogenic strains belong to phylogroups B2 and D^10^.

Investigating the bacterial population inhabiting the local water bodies is important because it reflects the bacterial clones perpetuating in the human population of the adjoining areas. Thus, *E. coli* from water bodies have been extensively investigated by several researchers and concerns over public-health risks associated with such contaminated water have been raised^11–22^. Moreover, water bodies can also act as genetic reactors in a manner similar to the host intestine, facilitating genetic exchange of virulence and antimicrobial resistance genes among different bacteria as these are often associated with mobile genetic elements like plasmids, transposons, insertion sequences etc^20,23,24^.

As per the latest records (retrieved in October 2022) 226,541 genome sequences of *E. coli* were present in the EnteroBase, of which 646 sequences were from strains isolated from India Supplementary Table 1. Deducing the phylogroups of these strains was difficult as the EnteroBase records did not specify their phylogroups and most of the original research papers describing these strains did not divulge their phylogroups. Thus, to the best of our knowledge this is the first report describing the genomic features of three *E. coli* phylogroup D strains isolated from India. The strains *E. coli* IP9, *E. coli* KKA and *E. coli* IPE were earlier isolated from the river Yamuna, a major anthropogenic urban river of India^25,26^. The 22 km stretch of the river that traverses through the National Capital Region of India from which these strains were isolated, receives effluents from industries, hospitals, local population etc. The antibiotic susceptibilities/resistance of these strains were determined by the standard disk diffusion assay and confirmed by gene sequencing of the relevant genes, and the results have been published^25,26^. Among these strains, *E. coli* IP9 was susceptible to all antibiotics tested, except ampicillin, *E. coli* KKA was a multi-β-lactam resistant strain^25,26^ and *E. coli* IPE was a multidrug resistant strain (resistant to multiple β-lactams, fluoroquinolones and kanamycin)^25,26^.

Despite the presence of a large number of genome sequences in the databases, it is very difficult to select the most appropriate genome as the template for the assembly. Hence, in the present study we have adopted a novel approach to assemble the whole genomes of these strains by combining both *de-novo* and reference-based approaches. Named as *de-novo* reference-based guided assembly, this approach helps the user in selecting the best reference genome from the public databases for a better-read assembly/contig scaffolding. Since these isolates showed varied antibiotic susceptibilities, we have also annotated their genomes to predict/identify genes for antimicrobial resistance and virulence factors. Also, we have provided Gene Ontology (GO) annotations that will be useful in analysing the role of individual genes.

## Methods

### DNA isolation, library preparation and sequencing

The three *E. coli* strains preserved as glycerol stocks (50% v/v) in a −80 °C deep refrigerator in our laboratory were revived by overnight incubation in LB broth at 37 °C, 200 rpm. Bacteria were grown up to the exponential phase (OD_600_ = 0.8) and harvested by centrifugation at 8000 rpm for 8 minutes at 4 °C. Genomic DNA was extracted using Nucleospin Microbial DNA kit (Macherey Nagel, Germany) and quantified by Qubit 4 fluorometer (Thermo Fisher Scientific, USA). These strains were isolated from the river Yamuna traversing through the National Capital Region of Delhi and identified/characterized by standard microbiological methods described earlier^25^. The DNA sequencing library was prepared by QIASeq FX DNA Library Kit (Qiagen, USA). Quantitative and qualitative library QC was done by Qubit 4 fluorometer (Thermo Fisher Scientific, USA) and TapeStation 4150 (Agilent technologies, USA), respectively. The libraries were sequenced on NovaSeq. 6000 (Illumina, USA) using 2 × 150 bp paired end reads with an insert size of 250–350 bp. The raw data statistics is shown in Table 1.

**Table 1:** Sequencing raw data statistics of *E. coli* strains.

Sample	Strain	Read orientation	Mean Phred Score	Number of reads	% GC	Mean read length (bp)
SAMPLE_A_2_FKDL210232083-1a_H722HDSX2_L2	IPE	R1	36.04	6,482,921	51	150
SAMPLE_A_2_FKDL210232083-1a_H722HDSX2_L2	IPE	R2	35.45	6,482,921	50	150
SAMPLE_B_DKDL210005881-1a_HFTKFDSX2_L3	KKA	R1	35.18	4,834,176	50	150
SAMPLE_B_DKDL210005881-1a_HFTKFDSX2_L3	KKA	R2	35.31	4,834,176	50	150
SAMPLE_C_DKDL210005882-1a_HFTKFDSX2_L3	IP9	R1	36.02	4,518,785	50	150
SAMPLE_C_DKDL210005882-1a_HFTKFDSX2_L3	IP9	R2	34.93	4,518,785	50	150

### Pre-processing and *de-novo* assembly: contig generation

Read quality was assessed using FastQC (v0.11.8) (https://www.bioinformatics.babraham.ac.uk/projects/fastqc/) and results were summarized using MultiQC (v1.13a) (https://multiqc.info/). The reads were trimmed using Trimmomatic^27^ (v0.39) at default parameters to remove Illumina adaptors, low-quality bases and/or reads less than 36 bp. The trimmed short overlapped paired-end reads obtained after Trimmomatic run were merged using FLASH^28^ (v1.2.11) at default parameters to create longer reads (single-end) for each isolate Table 2. The merged single-end reads along with the remaining trimmed pair-end reads were used to perform *de-novo* genome assembly using Unicycler^29^ (v0.4.8; *-m 200*). A total of 139, 100 and 183 contigs were generated after *de-novo* genome assembly for IP9, KKA and IPE, respectively. The N_50_ values for *E. coli* strains IP9, KKA and IPE were 129,988 bp, 178,741 bp and 252,171 bp, respectively, and were assessed using QUAST^30^ (v5.0.2).

**Table 2:** *E. coli* strains genome assembly statistics.

Genome assembly statistics	*E. coli* IP9	*E. coli* KKA	*E. coli* IPE
# Number of reads after Trimmomatic run	3,682,617	4,255,723	4,762,800
# Number of reads after Flash run	2,182,721 (extended) 1,499,896 (remaining)	2,251,378 (extended) 2,004,345 (remaining)	2,913,564 (extended) 1,849,236 (remaining)
# contigs generated	124	76	137
Total length (bp)	4,910,225	5,101,091	5,225,435
GC (%)	50.84%	50.55%	50.64%
Largest contig (bp)	491,679	476,221	720,451
N_50_ (bp)	137,460	283,877	295,668

### Selection of closest reference for scaffolding

The trimmed short paired-end reads obtained after Trimmomatic run were used to calculate the mash value against the 1875 *E. coli* complete genomes, downloaded from the NCBI database. Using plentyofbugs (https://github.com/nickp60/plentyofbugs) we identified the best reference genome for each strain. Then the trimmed reads of each strain were aligned against these respective best reference genomes using Bowtie 2^31^ and the percentage of alignment was calculated. The best reference genomes selected by the plentyofbugs for the three *E. coli* strains were different. For IP9 it was NZ_CP025859.1 (identity 77.60%), for KKA it was NZ_CP018206.1 (identity 96.34%) and for IPE it was NZ_CP046396.1 (85.39%).

### Closest reference-based scaffolding

**Fig. 1:**
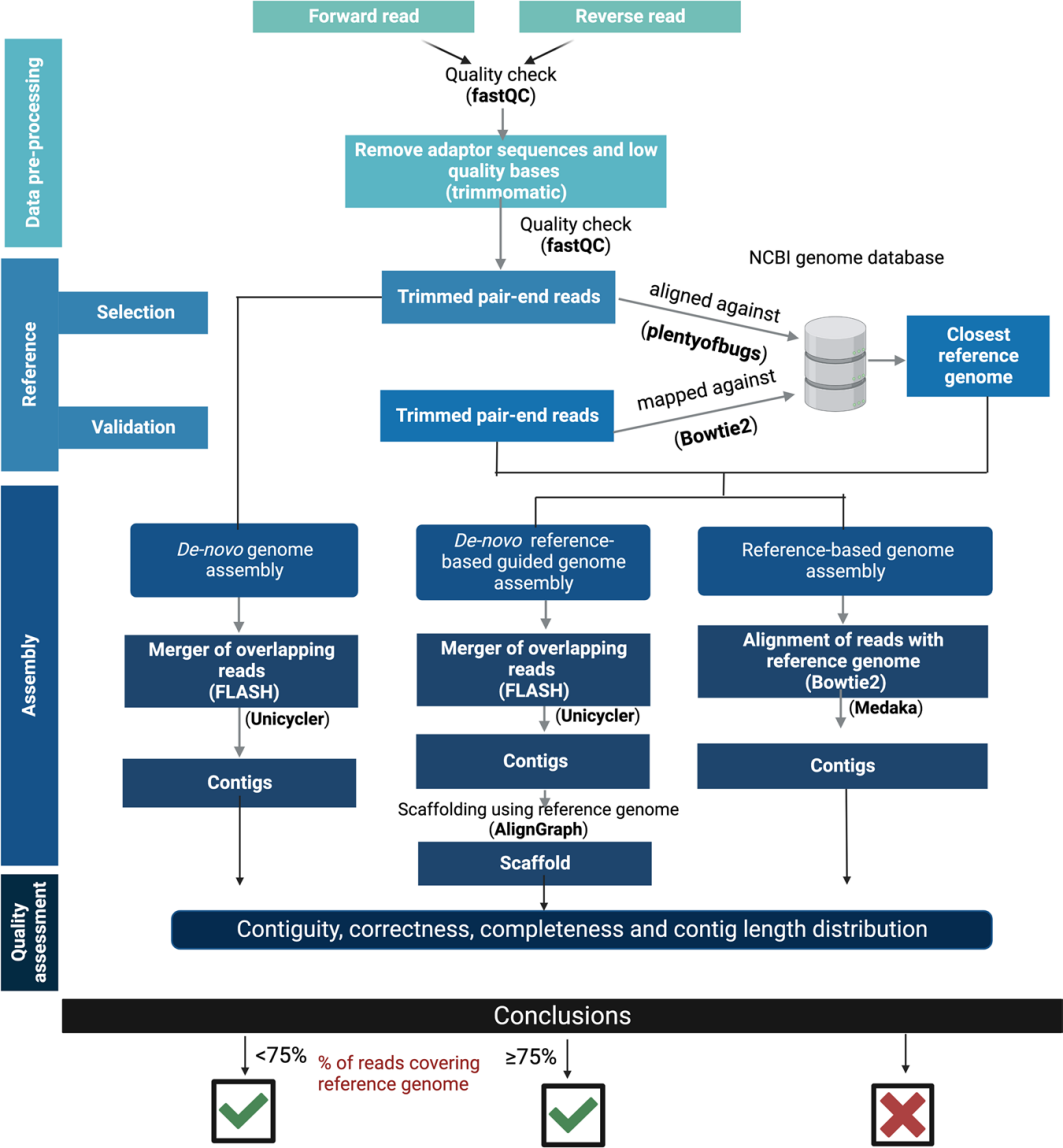
Schema of *de-novo* reference-based guided genome assembly.

**Fig. 2:**
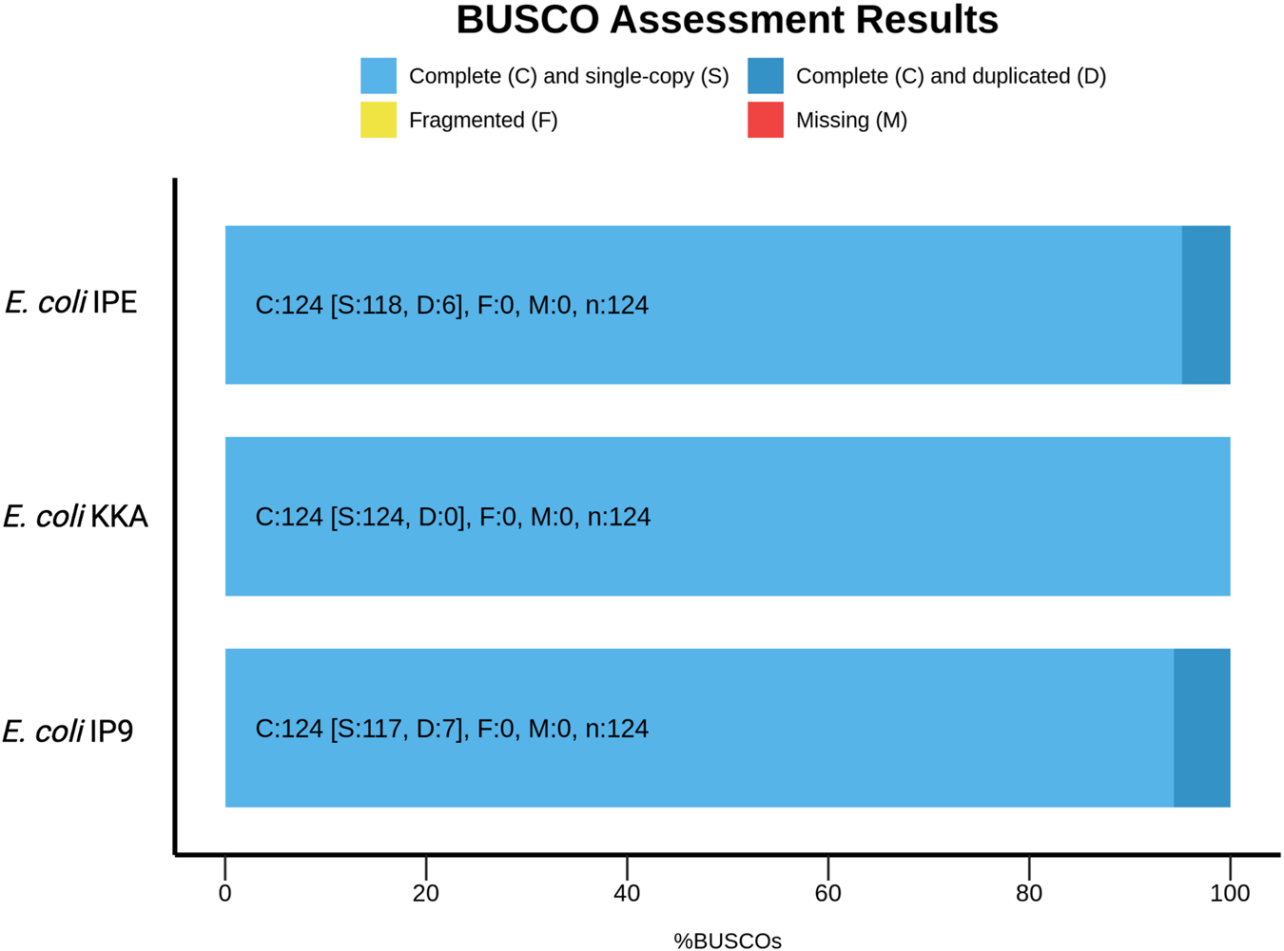
Quality assessment of the assembled genomes. BUSCO analysis showed 100% gene completeness in genomes of all the three strains, with no fragmented or missing gene orthologs.

*De-novo* assembled contigs of each isolate were scaffolded using their respective identified reference genome to fill the gaps between the assembled contigs using the Align-graph^32^ tool. The number of contigs generated after scaffolding were 124, 75 and 137 for IP9, KKA and IPE, respectively. The N_50_ values were assessed using QUAST (v5.0.2) which were 137,460 bp, 283,877 bp and 295,668 bp for *E. coli* strains IP9, KKA and IPE, respectively. The complete flow chart of the methodology is shown in Fig. 1. Further, BUSCO^33^ analysis revealed that the genomes showed 100% gene completeness Fig. 2.

### Genome annotations

The assembled genomes were annotated using the prokaryotic genome annotation pipeline Prokka^34^ (v1.14.6). Annotations revealed that the draft genomes of *E. coli* strains IP9 and KKA were 4,910,225 bp and 5,101,091 bp, respectively without any circular plasmid sequences. The draft chromosomal genome of *E. coli* strain IPE was 5,225,435 bp long and six complete circular plasmids of 5,165 bp (plasmid 1), 4,234 bp (plasmid 2), 4,072 bp (plasmid 3), 3,174 bp (plasmid 4), 2,101 bp (plasmid 5) and 1,459 bp (plasmid 6) were discerned using Unicycler (v0.4.8) with estimated multiplicities of 14.45x, 16.88x, 24.36x, 12.54x, 23.12x and 41.90x, respectively. Further characterization of these plasmids using the plasmid database PLSDB^35^ (v. 2021_06_23_v2) showed that Plasmid 1 (5165 bp) was similar to plasmid NZ_LR890613.1 with 99% identity and 47.53% GC content, plasmid 2 (4234 bp) with plasmid NZ_CP043038.1 with 99% sequence identity and 55.38% GC content, plasmid 3 (4072 bp) with plasmid NZ_CP023833.1 with 100% identity and 51.45% GC content, plasmid 4 (3174 bp) with plasmid NZ_CP031658.1 with 100% identity and 46.79% GC content, plasmid 5 (2101 bp) with plasmid CP045788.1 with 100% identity and 41.17% GC content and plasmid 6 (1459 bp) was similar to plasmid NZ_CP061061.1 with 100% identity and 50.93% GC content. In *E. coli* IP9, Prokka identified 124 contigs with 4,545 protein encoding sequences including 938 hypothetical proteins, 12 rRNAs, 82 tRNAs, 1 tmRNA and 24 pseudogenes that were present in 124 contigs. In *E. coli* KKA, it identified 76 contigs containing 4,772 protein encoding sequences including 1154 hypothetical proteins, 11 rRNA, 90 tRNA, 1 tmRNA and 20 pseudogenes. In *E. coli* IPE it identified 137 contigs with 4,934 protein encoding sequences including 1250 hypothetical proteins, 15 rRNAs, 86 tRNAs, 1 tmRNA and 20 pseudogenes. The GO annotation of the three *E. coli* strains IP9, KKA and IPE was also performed using the PANNZER2^36^ web service at default parameters. Of the 4772 proteins of IP9, PANNZER 2 web tool revealed 23,828 Gene Ontology (GO) annotations for 4127 proteins. In KKA, of the total 4545 total proteins, PANNZER 2 provided 22,794 GO annotations for 4193 proteins, and for IPE of the 4934 proteins it provided 23,185 GO annotations for 4381 proteins.

### Sequence type (ST), phylotyping, virulence factors and antimicrobial resistance genes identification

To identify the sequence type (ST), phylotyping of each strain was performed using EzClermont^37^ (v 0.7.0) and serotyping was done using ECTyper^38^. Virulence factors were checked using VFDB^39^ (Virulence Factors Database) using BLASTn (parameters: identity = 50%, query coverage = 50%, e-value = 1e-6). VFDB revealed the presence of genes encoding for virulence factors like adhesion, effector delivery system, exotoxin, invasion, mobility and nutritional/metabolic factors. Mobile genetic elements were found to be present in all the *E. coli* strains while genes for biofilm formation were found only in multi-β-lactam resistant and multidrug resistant strains. Several antimicrobial resistance genes were present only in multi-β-lactam resistant and multidrug resistant strains (KKA and IPE) but absent in the ampicillin resistant strain (IP9) as discerned using antibiotic resistance gene databases ResFinderdb^40^ at BLASTn parameters, identity = 75%, e-value = 1e-6. The presence of bacterial drug efflux pump genes was checked using BacEffluxPred^41^. The results have been summarized in Supplementary Table 2.

## Data Records

DNA sequencing data was submitted to NCBI Sequence Read Archive (SRA) database under the IDs: SRR21887619^42^, SRR21887618^43^ and SRR21887617^44^ and are associated with the NCBI BioProject accession number PRJNA890036. The genome assemblies are available under NCBI GenBank accession number GCA_026183935.1^45^, GCA_026183955.1^46^ and GCA_026183915.1^47^. The genome annotation and gene ontology files are publicly available at Figshare^48^.

## Technical Validation

### Optimization

**Fig. 3:**
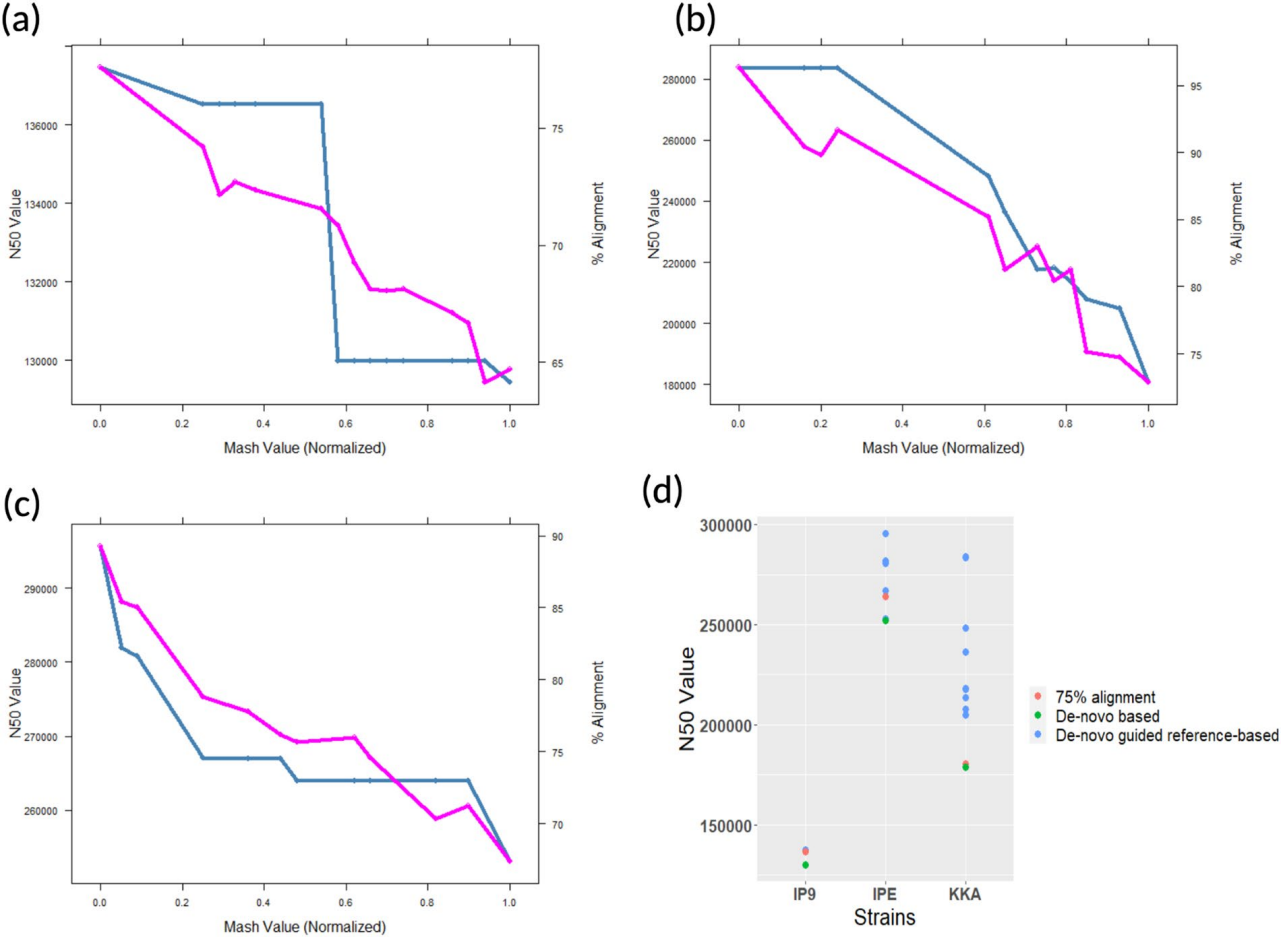
(**a**–**c**) Depiction of N_50_ values (blue colour) and percentage alignment (pink colour) of sequencing reads and reference genomes vis-à-vis Mash values discerned through the *de-novo* reference-based guided approach for *E. coli* strains **(a)** *E. coli* IP9 **(b)** *E. coli* KKA **(c)** *E. coli* IPE; **(d)** composite depiction of comparative N_50_ values of the three *E. coli* strains using *de-novo* reference-based guided assembly (magenta dots) and *de-novo* assembly (green dots). The orange dot represents the N_50_ value of the assembly when 75% of the sequence reads were aligned with reference genomes.

Improvement in the N_50_ values of the assemblies after reference-based scaffolding strongly indicated the benefits of our approach. To find the least identity percentage between the sample reads and the genome sequence in the database till which a genome sequence may be used as a template for scaffolding, we determined the N_50_ value of the assembled genomes before and after scaffolding at different identity percentages. For this, we calculated the Mash values between the sample reads and all the *E. coli* genomes available in the NCBI genome database using Mash tool^49^ (v2.3) at a default kmer size of 21. To reduce the time required to scaffold a large number of assembled genomes, the Mash value was normalized in the range of 0–1. Starting from 0, a sliding cut-off of 0.05 was used to select the genomic scaffold. Based on this, 16, 12 and 13 genomes were selected as references for IP9, KKA and IPE, respectively. We also aligned the sample reads with the selected *E. coli* genomes to calculate the % of alignment using Bowtie 2 and SAMtools^50^ (--flagstat). Initially, *de-novo* assembly of all the three sample reads were performed using Unicycler and then scaffolding was done using AlignGraph with all the selected genomes as reference. The N_50_ value before and after scaffolding was calculated using QUAST. Our results revealed that as the Mash value (i.e., distance) between the genome investigated and the references increased, the % of alignment and N_50_ both decreased^51^ Fig. 3a–c.

We also found that when sample reads were aligned with at least 75% of the reference genome, the N_50_ value after scaffolding was roughly equal to the *de-novo* N_50_ value^51^ Fig. 3d. This suggests that selection of the reference for scaffolding should be based on two considerations: (a) at least 75% of the sample reads should be aligned to the reference genome and (b) among all the available genomes in the database, the reference genome should have minimum Mash value.

### Benchmarking

To benchmark the performance of our approach vis-à-vis *de-novo* and reference-based assembly, we compared the N_50_ values, contig numbers and contig distribution of the assemblies obtained by all the three methods. Reference-based assembly was performed by aligning trimmed pair-end reads to the reference genome using Bowtie 2. On the basis of aligned reads, a consensus sequence was created using Medaka v1.3.3 (https://github.com/nanoporetech/medaka). The consensus sequences were considered as an assembled genome. In all the three genomes, the highest N_50_ values were discerned with the *de-novo* reference-based guided assembly approach *i.e*., 295,668 bp for IPE, 283,877 bp for KKA and 137,460 bp for IP9. The N_50_ values with the *de-novo* approach were 252,171 bp for IPE; 178,741 bp for KKA and 129,988 bp for IP9. The reference-based assembly gave the lowest N_50_ values *i.e*., 240,470 bp for IPE; 267,493 bp for KKA and 112,332 bp for IP9.

In context to the contig numbers, no correlation was observed between the number of contigs and the approach used for assembly. The number of contigs generated after *de-novo* reference-based guided assembly approach were 137 for IPE, 75 for KKA were and 124 for IP9. With the *de-novo* approach, the number of contigs were 183 for IPE, 100 for KKA and 139 for IP9. With the reference-based approach, the number of contigs were 85 for IPE, 74 for KKA and 461 for IP9.

**Fig. 4:**
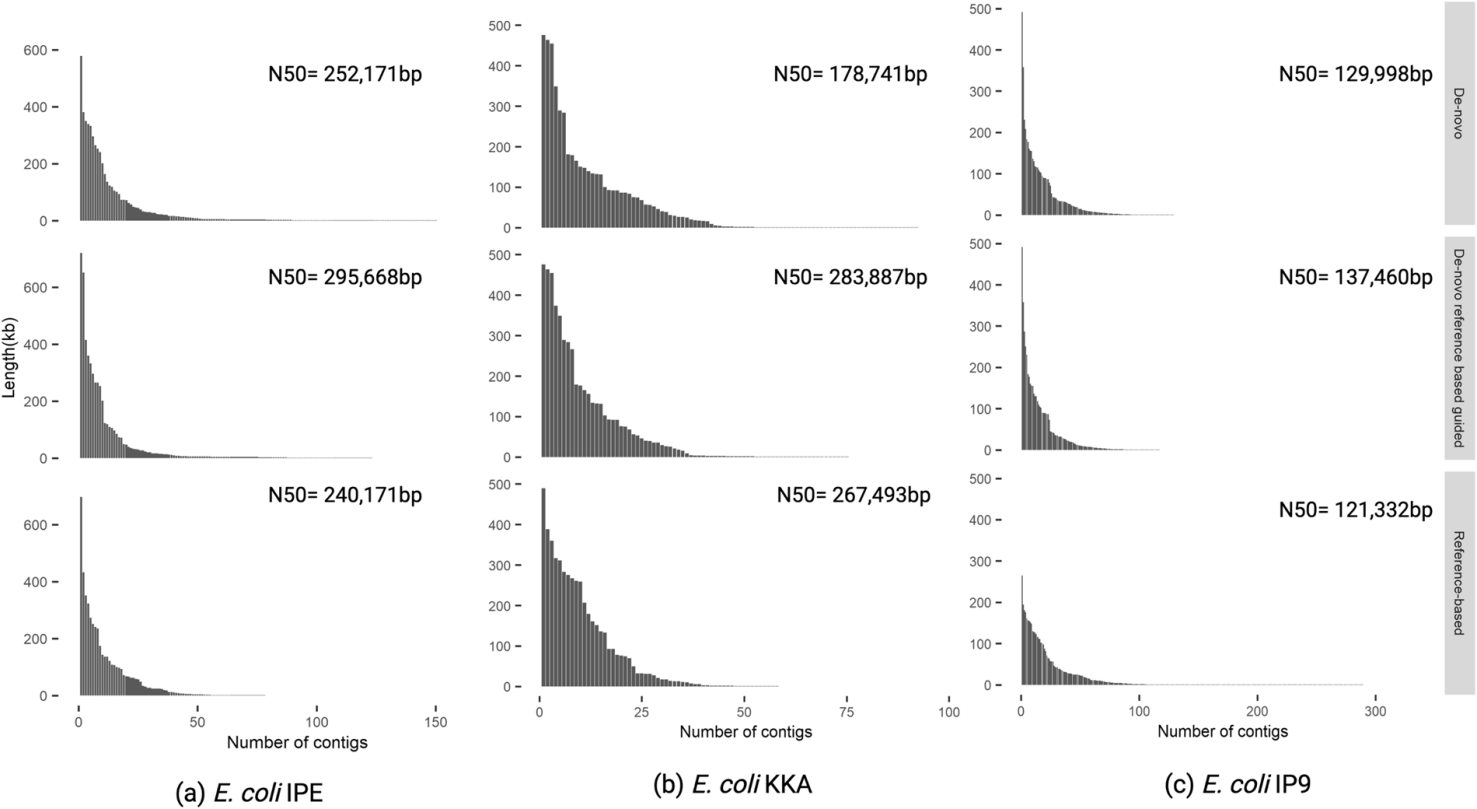
Contig distribution pattern plot depicts the contig length distribution, contig number and N_50_ value after *de-novo*, reference-based and *de-novo* reference-based guided approach for genome assemblies of (**a**) *E. coli* IPE (**b**) *E. coli* KKA (**c**) *E. coli* IP9.

Analysis of the contig lengths indicated that the length of the contigs with *de-novo* reference-based guided assembly approach was greater than the other two assembly approaches. Interestingly, with increase in the percentage identity of the investigated genome with the reference genome, lengths of the contigs also increased Fig. 4.

Overall, results of the benchmarking indicated that *de-novo* reference-based guided assembly approach gave a higher N_50_ value and contig length.

### Quality assessment (3 C’s) of the genome assemblies

#### Contiguity

Contiguity assessment of the three *E. coli* genome assemblies with our approach in comparison with the *de-novo* and reference-based assembly was done on the basis of the N_50_ values. The N_50_ value of each assembly was calculated using QUAST (QUality ASsessment Tool). The N_50_ values of the *E. coli* strains IP9, KKA and IPE using the *de-novo* approach were 129,988 bp, 178,741 bp and 252,171 bp, respectively. The N_50_ values of the *E. coli* strains IP9, KKA and IPE using reference-based approach were 112,332 bp, 267,493 bp and 240,470 bp, respectively. While, the N_50_ value of the *E. coli* strains IP9, KKA and IPE using *de-novo* reference-based guided approach were 137,460 bp, 283,877 bp and 295,668 bp, respectively. The maximum increment in N_50_ was recorded with the *de-novo* reference-based guided approach Supplementary Table 3.

#### Correctness

The correctness of the assembly was assessed by remapping the trimmed pair-end reads on the assembled genomes with all the three approaches using short read aligner Bowtie 2. The percentage (%) of alignment of reads with the *de-novo* assembly approach was 96.65% for IPE, 97.15% for KKA and 93.11% for IP9. The percentage (%) of alignment of the reads with the reference-based assembly approach was 84.43% for IPE, 96.05% for KKA and 77.75% for IP9. The percentage (%) of alignment of reads with the *de-novo* reference- based guided assembly was 96.88% for IPE, 97.47% for KKA and 93.41% for IP9. The correctness of the assembly was found to be maximum with the *de-novo* reference-based guided approach Supplementary Table 3.

#### Completeness

Assessment of completeness of genome assemblies of the three *E. coli* strains was done by BUSCO (Benchmarking Universal Single-copy Ortholog) using lineage dataset bacteria_odb10 (Creation date: 2020-03-06, number of genomes: 4085, number of BUSCOs: 124) and enterobacterales_odb10 (Creation date: 2021-02-23, number of genomes: 212, number of BUSCOs: 440). The assessment of BUSCO is based on the number of single-copy orthologs that are shared among the newly assembled genome and higher taxonomic groups. A high fraction of complete BUSCOs indicates completeness of assembly while a high proportion of missing and fragmented BUSCOs indicates incompleteness of the assembly. Using bacteria_odb10 124 BUSCOs and with enterobacterales_odb10, 440 BUSCOs were discerned in the three genomes assemblies and none was found to be fragmented or missing with the *de-novo* reference-based guided approach. Similarly, none of the genome assemblies was fragmented or missing using the *de-novo* approach. Though IP9 and KKA genome assemblies were complete, IPE assembly was found to be fragmented with the reference-based approach Supplementary Table 3. This indicated that the *de-novo* reference-based guided and *de-novo* approaches give complete genome assemblies than the reference-based approach. The evaluation of completeness of the 16S rRNA genes of all the strains by the three assembly approaches by BLAST against the 16S rRNA database (NCBI) revealed ≥99% alignment in the *E. coli* 16S rRNA sequences present in the database and the assemblies.

The technical checks for assessing the quality of the assembled genomes like, N_50_ value, BUSCO, % of read alignment and reference genome selection using plentyofbugs suggests that genome assembly using the *de-novo* reference-based guided assembly is better than *de-novo* assembly and reference- based assemblies.

The contig distribution pattern based upon the contig number and the length of each contig suggested that the selection of approach for genome assembly should be based upon two considerations: (a) when the identity between the reference and the reads is less than 75%, both *de-novo* and *de-novo* reference-based guided assembly approaches are good, because both give similar N_50_ values. However, the reference-based approach is unsuitable in these cases, as it gives a distorted assembly with a very high contig number and very low N_50_ value. (b) when the identity between the reference and the reads is more than or equal to 75%, *de-novo* reference-based guided assembly is better than *de-novo* and reference-based assemblies because it gives better contiguity (N_50_ values), completeness and correctness of the assembly Fig. 4.

## Usage Notes

The assembly method was built using the well-known genome assemblers and tools hence, can be easily integrated in any genome analysis workflow. Further, all the software commands have been compiled under one wrapper code, making it convenient and user-friendly for analysing prokaryotic genomes. We believe that the methods described here would help the scientific community in faster/better assembly using short read sequencing.

### Supplementary information

Supplementary Table 1

Supplementary Table 2

Supplementary Table 3

## Data Availability

The output files of technical validation^51^ and the codes for *de-novo* reference-based guided assembly^52^ is available on figshare.

## References

[1] Wirth T (2006). Sex and virulence in Escherichia coli: an evolutionary perspective. Mol. Microbiol..

[2] Blount ZD (2015). The unexhausted potential of E. coli. eLife.

[3] Berthe T, Ratajczak M, Clermont O, Denamur E, Petit F (2013). Evidence for Coexistence of Distinct Escherichia coli Populations in Various Aquatic Environments and Their Survival in Estuary Water. Appl. Environ. Microbiol..

[4] Lopez-Torres AJ, Hazen TC, Toranzos GA (1987). Distribution and *in situ* survival and activity ofKlebsiella pneumoniae andEscherichia coli in a tropical rain forest watershed. Curr. Microbiol..

[5] Ishii S, Sadowsky MJ (2008). Escherichia coli in the Environment: Implications for Water Quality and Human Health. Microbes Environ..

[6] Texier S (2008). Persistence of Culturable Escherichia coli Fecal Contaminants in Dairy Alpine Grassland Soils. J. Environ. Qual..

[7] Brennan FP, O’Flaherty V, Kramers G, Grant J, Richards KG (2010). Long-Term Persistence and Leaching of Escherichia coli in Temperate Maritime Soils. Appl. Environ. Microbiol..

[8] de los Angeles Dublan M, Ortiz-Marquez JCF, Lett L, Curatti L (2014). Plant-Adapted Escherichia coli Show Increased Lettuce Colonizing Ability, Resistance to Oxidative Stress and Chemotactic Response. PLOS ONE.

[9] Walk ST (2009). Cryptic Lineages of the Genus Escherichia. Appl. Environ. Microbiol..

[10] Picard B (1999). The link between phylogeny and virulence in Escherichia coli extraintestinal infection. Infect. Immun..

[11] US EPA, O. National Recommended Water Quality Criteria Tables. https://www.epa.gov/wqc/national-recommended-water-quality-criteria-tables (2014).

[12] Duran M, Haznedaroğlu BZ, Zitomer DH (2006). Microbial source tracking using host specific FAME profiles of fecal coliforms. Water Res..

[13] Jiang SC (2007). Microbial source tracking in a small southern California urban watershed indicates wild animals and growth as the source of fecal bacteria. Appl. Microbiol. Biotechnol..

[14] Kaneene JB (2007). Considerations When Using Discriminant Function Analysis of Antimicrobial Resistance Profiles To Identify Sources of Fecal Contamination of Surface Water in Michigan. Appl. Environ. Microbiol..

[15] Ahmed W, Goonetilleke A, Powell D, Chauhan K, Gardner T (2009). Comparison of molecular markers to detect fresh sewage in environmental waters. Water Res..

[16] Fremaux B, Gritzfeld J, Boa T, Yost CK (2009). Evaluation of host-specific Bacteroidales 16S rRNA gene markers as a complementary tool for detecting fecal pollution in a prairie watershed. Water Res..

[17] Silkie SS, Nelson KL (2009). Concentrations of host-specific and generic fecal markers measured by quantitative PCR in raw sewage and fresh animal feces. Water Res..

[18] Kelty CA, Varma M, Sivaganesan M, Haugland RA, Shanks OC (2012). Distribution of Genetic Marker Concentrations for Fecal Indicator Bacteria in Sewage and Animal Feces. Appl. Environ. Microbiol..

[19] Singhal N, Singh NS, Maurya AK, Virdi JS (2019). Virulence-associated traits and *in vitro* biofilm-forming ability of Escherichia coli isolated from a major river traversing Northern India. Environ. Sci. Pollut. Res..

[20] Singh, S., Singh, S. K., Chowdhury, I. & Singh, R. Understanding the Mechanism of Bacterial Biofilms Resistance to Antimicrobial Agents. *Open Microbiol. J*. **11**, (2017). 10.2174/1874285801711010053 PMC5427689 28553416

[21] Dhanji H, Doumith M, Hope R, Livermore DM, Woodford N (2011). ISEcp1-mediated transposition of linked blaCTX-M-3 and blaTEM-1b from the IncI1 plasmid pEK204 found in clinical isolates of Escherichia coli from Belfast, UK. J. Antimicrob. Chemother..

[22] Walsh TR, Weeks J, Livermore DM, Toleman MA (2011). Dissemination of NDM-1 positive bacteria in the New Delhi environment and its implications for human health: an environmental point prevalence study. Lancet Infect. Dis..

[23] Baquero F, Martínez J-L, Cantón R (2008). Antibiotics and antibiotic resistance in water environments. Curr. Opin. Biotechnol..

[24] Wellington EMH (2013). The role of the natural environment in the emergence of antibiotic resistance in gram-negative bacteria. Lancet Infect. Dis..

[25] Bajaj P, Singh NS, Kanaujia PK, Virdi JS (2015). Distribution and molecular characterization of genes encoding CTX-M and AmpC β-lactamases in Escherichia coli isolated from an Indian urban aquatic environment. Sci. Total Environ..

[26] Singh NS, Singhal N, Kumar M, Virdi JS (2022). Public health implications of plasmid-mediated quinolone and aminoglycoside resistance genes in Escherichia coli inhabiting a major anthropogenic river of India. Epidemiol Infect..

[27] Bolger AM, Lohse M, Usadel B (2014). Trimmomatic: a flexible trimmer for Illumina sequence data. Bioinformatics.

[28] Magoč T, Salzberg SL (2011). FLASH: fast length adjustment of short reads to improve genome assemblies. Bioinformatics.

[29] Wick RR, Judd LM, Gorrie CL, Holt KE (2017). Unicycler: Resolving bacterial genome assemblies from short and long sequencing reads. PLOS Comput. Biol..

[30] Gurevich A, Saveliev V, Vyahhi N, Tesler G (2013). QUAST: quality assessment tool for genome assemblies. Bioinformatics.

[31] Langmead B, Salzberg SL (2012). Fast gapped-read alignment with Bowtie 2. Nat. Methods.

[32] Bao E, Jiang T, Girke T (2014). AlignGraph: algorithm for secondary de novo genome assembly guided by closely related references. Bioinformatics.

[33] Simão FA, Waterhouse RM, Ioannidis P, Kriventseva EV, Zdobnov EM (2015). BUSCO: assessing genome assembly and annotation completeness with single-copy orthologs. Bioinformatics.

[34] Seemann T (2014). Prokka: rapid prokaryotic genome annotation. Bioinformatics.

[35] Galata V, Fehlmann T, Backes C, Keller A (2019). PLSDB: a resource of complete bacterial plasmids. Nucleic Acids Res..

[36] Törönen P, Medlar A, Holm L (2018). PANNZER2: a rapid functional annotation web server. Nucleic Acids Res..

[37] Waters NR, Abram F, Brennan F, Holmes A, Pritchard L (2020). Easy phylotyping of Escherichia coli via the EzClermont web app and command-line tool. Access Microbiol..

[38] Bessonov K (2021). ECTyper: in silico Escherichia coli serotype and species prediction from raw and assembled whole-genome sequence data. Microb. Genomics.

[39] Chen L (2005). VFDB: a reference database for bacterial virulence factors. Nucleic Acids Res..

[40] Florensa AF, Kaas RS, Clausen PTLC, Aytan-Aktug D, Aarestrup FM (2022). ResFinder – an open online resource for identification of antimicrobial resistance genes in next-generation sequencing data and prediction of phenotypes from genotypes. Microb. Genomics.

[41] Pandey D, Kumari B, Singhal N, Kumar M (2020). BacEffluxPred: A two-tier system to predict and categorize bacterial efflux mediated antibiotic resistance proteins. Sci. Rep..

[42] (2022). NCBI Sequence Read Archive.

[43] (2022). NCBI Sequence Read Archive.

[44] (2022). NCBI Sequence Read Archive.

[45] Aswal M, Singhal N, Kumar M (2022). GenBank..

[46] Aswal M, Singhal N, Kumar M (2022). GenBank..

[47] Aswal M, Singhal N, Kumar M (2022). GenBank..

[48] Kumar M, Aswal M, Singhal N (2023). Figshare.

[49] Ondov BD (2016). Mash: fast genome and metagenome distance estimation using MinHash. Genome Biol.

[50] Li H (2009). The Sequence Alignment/Map format and SAMtools. Bioinforma. Oxf. Engl..

[51] Kumar M, Aswal M, Singhal N (2023). Figshare.

[52] Kumar M, Singhal N, Aswal M (2022). Figshare.

